# Complexity of Recent Earthquake Swarms in Greece in Terms of Non-Extensive Statistical Physics

**DOI:** 10.3390/e25040667

**Published:** 2023-04-16

**Authors:** Eirini Sardeli, Georgios Michas, Kyriaki Pavlou, Filippos Vallianatos, Andreas Karakonstantis, Georgios Chatzopoulos

**Affiliations:** 1 Section of Geophysics-Geothermics, Department of Geology and Geoenvironment, National and Kapodistrian University of Athens, 15784 Athens, Greece; 2 Institute of Physics of Earth’s Interior and Geohazards, UNESCO Chair on Solid Earth Physics and Geohazards Risk Reduction, Hellenic Mediterranean University Research & Innovation Center, 73133 Chania, Greece

**Keywords:** Tsallis entropy, complexity, Non-Extensive Statistical Physics, earthquake swarms, Greece

## Abstract

Greece exhibits the highest seismic activity in Europe, manifested in intense seismicity with large magnitude events and frequent earthquake swarms. In the present work, we analyzed the spatiotemporal properties of recent earthquake swarms that occurred in the broader area of Greece using the Non-Extensive Statistical Physics (NESP) framework, which appears suitable for studying complex systems. The behavior of complex systems, where multifractality and strong correlations among the elements of the system exist, as in tectonic and volcanic environments, can adequately be described by Tsallis entropy (*S_q_*), introducing the *Q*-exponential function and the entropic parameter *q* that expresses the degree of non-additivity of the system. Herein, we focus the analysis on the 2007 Trichonis Lake, the 2016 Western Crete, the 2021–2022 Nisyros, the 2021–2022 Thiva and the 2022 Pagasetic Gulf earthquake swarms. Using the seismicity catalogs for each swarm, we investigate the inter-event time (*T*) and distance (*D*) distributions with the *Q*-exponential function, providing the *q_T_* and *q_D_* entropic parameters. The results show that *q_T_* varies from 1.44 to 1.58, whereas *q_D_* ranges from 0.46 to 0.75 for the inter-event time and distance distributions, respectively. Furthermore, we describe the frequency–magnitude distributions with the Gutenberg–Richter scaling relation and the fragment–asperity model of earthquake interactions derived within the NESP framework. The results of the analysis indicate that the statistical properties of earthquake swarms can be successfully reproduced by means of NESP and confirm the complexity and non-additivity of the spatiotemporal evolution of seismicity. Finally, the superstatistics approach, which is closely connected to NESP and is based on a superposition of ordinary local equilibrium statistical mechanics, is further used to discuss the temporal patterns of the earthquake evolution during the swarms.

## 1. Introduction

The clustering of earthquakes in both time and space without a prominent large-magnitude earthquake is commonly referred to as an earthquake swarm. Swarms can last for several days, weeks or months, registering many earthquakes within a small volume. According to [1], when the stress gradually increases for a variety of reasons, earthquake swarms occur at pre-existing cracked regions. The local fractures tend to break and cause small-magnitude earthquakes, instead of generating a strong fracture or rupture, inducing a larger-magnitude event. Numerous studies [2,3,4,5,6,7,8,9] suggest that earthquake swarms are either triggered by dynamic stress transfer effects caused by previous strong events, or by aseismic factors, such as aseismic creep and the intrusion of fluids into fracture zones linked with volcanic activity or pore fluid pressure diffusion. Swarm earthquakes have small magnitudes in the range of *M* 1.0 to *M* 5.0 and rarely produce earthquakes with large magnitudes, i.e., *M* ≥ 6 [10]. In these cases, the region releases strain energy instantly with these small magnitude earthquakes, rather than being under stress for a long time and preparing for a large magnitude earthquake. 

**Figure 1:**
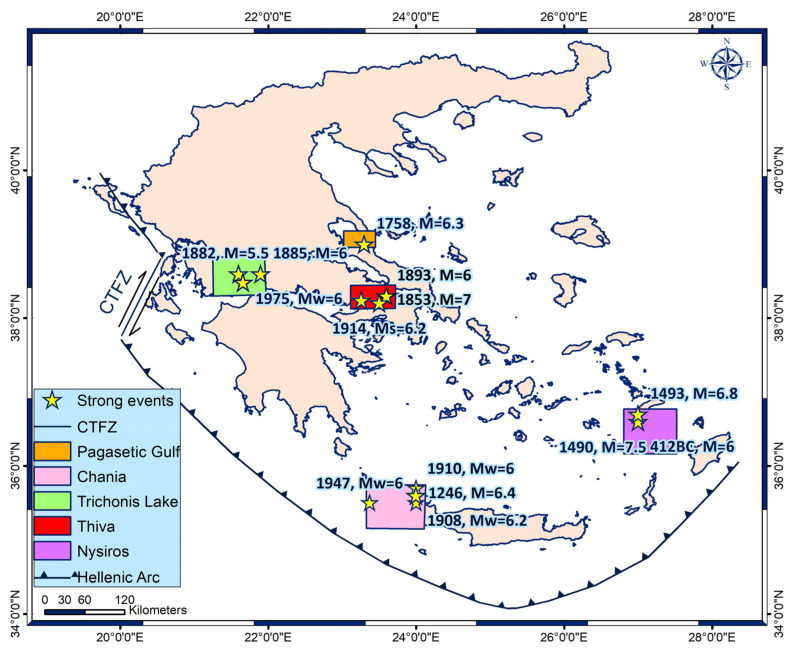
Geographical distribution of the five studied earthquake swarms in Greece. In each square, the stars represent the strong events that have taken place in the area, after [14]. The line with triangles (in black) represents the Hellenic Arc, which is a subduction zone of about 1000 km, where the African lithosphere is subducting under the Aegean lithospheric plate in a roughly SW–NE direction [15] and the black line indicates the Cephalonia Transform Fault Zone (CTFZ) [16].

Greece is located on a tectonically active plate boundary at the convergence of the Eurasian and African lithospheric plates south of Crete, forming the Hellenic arc (Figure 1). The Hellenic arc is formed by the outer sedimentary arc and the inner volcanic arc (Sousaki, Methana, Milos, Santorini and Nisyros). The active stress field in Greece is complex, as it generally switches from extension to compression from north to south. Greece and the adjacent areas (the Aegean Sea and western Turkey) are tectonically very active, presenting high seismic activity [11], the highest in Europe, while it ranks highly (sixth) on a global scale [12]. For this reason, it often hosts earthquakes of large magnitude, whilst a moderate or small magnitude earthquake is felt every 2–3 days on average. Most of these earthquakes are shallow, with some events being devastating for the human environment or for life losses (e.g., the 1881 Chios, 1894 Atalanti, 1953 Cephalonia, 1999 Athens, 2017 Kos, 2020 Samos earthquakes, etc.) [13].

Apart from the background seismic activity and the occurrence of strong events accompanied by pronounced aftershock sequences, frequent earthquake swarms also occur in the area of Greece. Some characteristic cases come from the active continental Corinth Rift, such as those of the 2001 Agios Ioannis swarm [17,18], the 2003–2004 swarm in the offshore region of the West Gulf of Corinth [19], the 2013 Helike [20,21], the 2015 Malamata [22] and the 2020 Perachora [7] swarms. Other cases are associated with active volcanoes, such as the 2011–2012 unrest at the Santorini caldera with swarms of micro-earthquakes [23,24], or CO_2_ emissions as the 2012–2014 microseismic activity in Florina [25,26]. Furthermore, a seismic excitation occurred in 2019 in the offshore area north of Lefkada Island, with more than 250 located events forming a small swarm [27].

In the present work, we study the physical characteristics of recent earthquake swarms that have occurred in the broader area of Greece and in different seismotectonic settings, such as the earthquake swarms of Trichonis Lake (8 April 2007–2 July 2007), Western Crete (1 February 2016–25 March 2016), Nisyros (7 April 2021–30 June 2022), Thiva (10 July 2021–1 July 2022) and Pagasetic Gulf (9 May 2022–23 June 2022) (Figure 1). Particularly, we investigate the scaling properties of the frequency–magnitude distribution (FMD) and of the spatiotemporal evolution of seismicity. The FMD is usually expressed in terms of the Gutenberg–Richter scaling relation [28]. Herein, we further describe the FMD with the fragment–asperity model [29] derived in the framework of Non-Extensive Statistical Physics (NESP) [30,31]. NESP, as developed by Tsallis [32], provides a generalization of the Boltzmann–Gibbs (BG) statistical physics and constitutes a suitable framework for studying complex systems exhibiting scale invariance, multi-fractality and long-range interactions [33]. Using NESP and based on the universal principle of entropy, we further describe the spatiotemporal scaling properties of the swarms using suitable scaling functions. Within this framework, we analyze the inter-event time and distance distributions of each earthquake swarm, fitting the observed data with the *Q*-exponential function in agreement with NESP, as proposed in a number of cases [34,35,36] for the spatiotemporal properties of seismicity in California and Japan, for aftershock sequences [37,38,39,40] and for global seismicity [41]. This study describes the energy and the spatiotemporal patterns of each earthquake swarm, providing the non-additive entropic parameters (*q_T_*, *q_D_*, *q_M_*). We demonstrate that NESP is an adequate and methodological tool for analyzing complex systems, such as the spatiotemporal evolution and the FMD of earthquake swarms. In addition, based on the observed scaling properties of seismicity, we describe the temporal evolution of the swarms using superstatistics [42,43,44] that complement the NESP approach.

## 2. A Non-Extensive Statistical Physics Approach (NESP)

### 2.1. Spatiotemporal Scaling Properties of Earthquake Swarms

Non-extensive statistical physics (NESP) is a generalization of Boltzmann–Gibbs (BG) statistical physics and has been used to describe complex dynamic systems that exhibit scale-invariance, (multi)fractality, long-range interactions and long-term memory effects, leading to broad distributions with power-law asymptotic behavior [32,45,46,47].

In 1988 [45,46], Tsallis proposed the non-additive Tsallis entropy (*Sq*) which is expressed as:

(1) $$S_{q}=k_{B}\frac{1-\sum p^{q}\left(X\right)}{q-1}$$

where *k_B_* is Boltzmann’s constant and *q* the entropic index that signifies the non-extensivity of the system. In the following, we address the NESP theory for a continuous variable *X* that may express the inter-event times (*T*), i.e., the time intervals between the successive seismic events, or the inter-event distances (*D*), i.e., the three-dimensional Euclidean distance between the foci of successive seismic events. 

In the case of *q* = 1, then *Sq* = *S_BG_* and the approach reduces to well-known Boltzmann–Gibbs (BG) entropy. Despite the fact that *Sq* and *S_BG_* have many similar properties in common, such as non-negativity, expansibility and concavity, there is a distinctive difference between the two entropies. The Boltzmann–Gibbs entropy is additive, meaning that the entropy of a combined system is the sum of the entropy of the separated parts, whereas the Tsallis entropy *Sq* is non-additive. According to this property and for two probabilistic independent events *A* and *B*, the total entropy *Sq* of the system *A* + *B* satisfies:

(2) $$\frac{S_{q\;}\left(A+B\right)}{k_{B}}=\frac{S_{q}\left(A\right)}{k_{B}}+\frac{S_{q}\left(B\right)}{k_{B}}+\left(1-q\right)\frac{S_{q}\left(A\right)}{k_{B}}\frac{S_{q}\left(B\right)}{k_{B}}$$

This property is known as pseudo-additivity and is further distinguished into super-additivity if *q* < 1, sub-additivity if *q* > 1 and additivity when *q* = 1 (Boltzmann–Gibbs statistics) where the last term on the right-hand side of Equation (2) vanishes, and the additivity property is recovered. This has been recently discussed in terms of the entropy defect [48].

For seismic events, the probability distribution *p*(*X*) of the continuous variable *X* (i.e., the inter-event times T or the inter-event distances *D*) is acquired by maximizing the non-extensive entropy *Sq* under appropriate constraints [46] using the Lagrange multipliers method that leads to the physical probability:

(3) $$p\left(X\right)=\frac{{\left[1-\left(1-q\right)\left(\frac{X}{X_{q}}\right)\right]}^{\frac{1}{1-q}}}{Z_{q}}=\frac{\exp_{q}\left(-\frac{X}{X_{q}}\right)}{Z_{q}}$$

where *Zq* refers to the *q*-partition function defined as:

(4) $$Z_{q}=\int_{0}^{\infty }\exp_{q}\left(-\frac{X}{X_{q}}\right)dX$$

and *Xq* is a generalized scaled inter-event time or inter-event distance, while the *q*-exponential function is defined as:

(5) $$\exp_{q}\left(X\right)={\left[1+\left(1-q\right)X\right]}^{\frac{1}{1-q}}$$

for $1+\left(1-q\right)X\geq 0$ and in all other cases $\exp_{q}\left(X\right)=0$ [46].

The corresponding to Equation (3) cumulative distribution function (*CDF*) *P* (>*X*) should be obtained upon integration of the escort probability distribution *P_q_* (*X*) [35,46,47,49]

(6) $$P\left(>X\right)=\int_{0}^{\infty }P_{q}\left(X\right)dX=\exp_{q}\left(-\frac{X}{X_{q}}\right)$$

If *P* (>*X*) is estimated by the integration of physical probability *p*(*X*) (Equation (3)) instead of the escort probability distribution *P_q_* (*X*), then:

(7) $$P\left(>X\right)={\left[1-\left(1-Q\right)\left(\frac{X}{X_{0}}\right)\right]}^{\frac{1}{1-Q}}=\exp_{Q}\left(-\frac{X}{X_{0}}\right)$$

that mathematically is the *Q*-exponential function, defined for *q* < 2, with $X_{0}=X_{q}Q$ ($X_{0}\;$ is a positive scaling parameter) and $Q=1/\left(2-q\right)$ or $q=2-\left(1/Q\right)$ [46,50,51,52,53]. The different forms are all correct and can be transformed one into the other by means of simple algebraic operations involving the values of *q* and *X*_0_. Moreover, the variable *X* refers to the inter-event times (*T*) or distances (*D*), while the *q*-value describes the spatiotemporal evolution and the degree of correlations, with *q_T_* > 1, for the inter-event times, and *q_D_* < 1 for the inter-event distances, respectively, according to [34,35]. 

The inverse of the *Q-*exponential function (Equation (7) for *X* > 0) leads to the *Q-*logarithmic function [46,49,54]:

(8) $$\ln_{Q}P\left(>X\right)=\frac{P{\left(>X\right)}^{1-Q}-1}{1-Q}=-\frac{1}{X_{0}}X$$

For *q* > 1, the *Q-*exponential function presents asymptotic power*–*law behavior, while for *q* < 1 a cut*-*off appears in the tail of the distribution. In the limit when *q* = 1, the *Q*-exponential and the *Q*-logarithmic recover the ordinary exponential and logarithmic expressions, respectively.

The latter Equation implies that after the estimation of the appropriate *q*-value that describes the distribution of *X*, the *Q*-logarithmic function (Equation (8)) is a straight line, with slope −1/*X*_0_ [54].

### 2.2. Frequency-Magnitude Distribution and Seismic b-Values

The Gutenberg–Richter scaling relation [28] is one of the most well-known empirical scaling relations in geophysics, which expresses the frequency of earthquake magnitudes in a specific area and has the following form:

(9) $$\log N\left(>M\right)=a-bM$$

where *M* represents the earthquake magnitude, *N*(>*M*) is the cumulative number of earthquakes with a magnitude equal to or greater than *M* and *a*, *b* are positive scaling parameters. Parameter *a* describes the regional level of seismicity and shows significant variations from one area to another. Parameter *b*, also known as the *b*-value, is the slope of the frequency–magnitude distribution (FMD) and describes the size distribution of the earthquake events. The estimated *b*-value usually has typical values close to 1 [55,56,57], especially for tectonic earthquakes, but there have been reported high *b*-values (up to 3) associated with volcano areas [58].

### 2.3. The Fragment–Asperity Model for Seismic Energies

Sotolongo-Costa and Posadas [29] developed the fragment*–*asperity model of earthquake interactions, which describes the earthquake generation mechanism within the NESP context. This model takes into account the interaction of two rough profiles (fault blocks) and the fragments filling the area in between them, originating from the local breakage of the tectonic plates. This interaction explains how earthquakes are triggered. These authors [29] applied the NESP formalism to estimate the seismic energy distribution function based on the size distribution of fragments and presented an energy distribution function, which contained the Gutenberg–Richter (G-R) scaling relation as a particular case. The fragment*–*asperity model has been used in a variety of applications, including regional and local seismicity, as well as volcanic seismicity [59,60,61].

Telesca [62] revised this model by considering that the magnitude (*M*) is related to the relative seismic energy and by taking into account the threshold magnitude *M_c_* [63], proposed a modified expression that relates the cumulative number of earthquakes with magnitude, given as [49]:

(10) $$\log\left(\frac{N(>M)}{N}\right)=\frac{2-q_{M}}{1-q_{M}}\log\left(\frac{1-\left(\frac{1-q_{M}}{2-q_{M}}\right)\left(\frac{10^{M}}{A^{2/3}}\right)}{1-\left(\frac{1-q_{M}}{2-q_{M}}\right)\left(\frac{10^{Mc}}{A^{2/3}}\right)}\right)$$

where *M* is the earthquake magnitude, *M_c_* is the threshold magnitude, *A* is proportional to the volumetric energy density and *q_M_* is the entropic index [61]. Temporal variations and the increase in *q_M_* indicate that the physical state of a seismic area moves away from equilibrium [64].

In contrast to the G-R scaling relation, the fragment–asperity model accurately describes the observed earthquake magnitudes across a wider range of scales, whereas for values above a certain threshold magnitude, the G-R relation can be derived as a special case, for the value of [49]:

(11) $$b=\frac{2-q_{M}}{q_{M}-1}$$

Moreover, the *b*-value calculation using the maximum-likelihood approach (e.g.**,** [65]) is rather sensitive to the initial selection of the minimum earthquake magnitude *M*_0_ in the catalogue of events, while the *q_M_*-value estimation is relatively stable irrespective of the selection of *M*_0_ [66].

## 3. Recent Earthquake Swarms in Greece 

In the present work, we analyze the spatiotemporal scaling properties and the frequency–magnitude distribution of earthquake swarms in terms of Tsallis Entropy. The location of the five earthquake swarms that we study in this work are illustrated in Figure 1. The areas of each swarm, depicted with colored squares, are also shown the strong events that have been reported with magnitude *M* ≥ 5.5 [14]. The study areas, as symbolized in Figure 1, are Thiva (red square), Nisyros island (purple square), Trichonis Lake (green square), Western Crete (pink square) and Pagasetic Gulf (orange square). The earthquake swarms are described chronologically from the oldest to the most recent. 

### 3.1. Seismotectonic Setting and Earthquake Datasets

#### 3.1.1. The 2007 Trichonis Lake Earthquake Swarm

Trichonis is the largest natural lake in western Greece which strikes WNW–ESE for a distance of about 32 km and has a width of about 10 km [67]. More specifically, it is situated in the eastern part of Aitoloakarnania and southeast of the city of Agrinio. Trichonis Lake is a late Plio-Quaternary extensional basin, created by back-arc extensional faulting in western Greece [68]. The basin containing Trichonis Lake is marked by a major north-dipping normal fault system which bounds the south shore of the lake [67]. The majority of the seismic events in the area are well-constrained along the southeastern side of the lake. Historical records exist since 1841 for that region, but since 1966, the seismic activity has been decreasing [69]. The most recent instrumentally recorded seismic sequence occurred during June–December 1975 near the southern flank of Lake Trichonis. The first of the strongest earthquakes took place on 30 June 1975 (*M_w_* 5.6), whereas on 21 December 1975 an *M_w_* 5.5 event was followed by another one on 31 December 1975 with a magnitude of *M_w_* 6.0 [69]. Other strong events in the area occurred in 1882 and 1885, with magnitudes of 5.5 and 6, respectively [14].

In the southeastern part of the lake, an intense seismic sequence, with a series of relatively strong earthquakes, started in April 2007. The sequence initiated with small events on 8 April and two days later the three strongest events of the entire sequence occurred. More analytically, on 10 April three strong earthquakes occurred at 03:17:56, 07:13:03 and 10:41:00 UTC with magnitudes *M_w_* 4.9, 4.9 and 5.2, respectively. The seismic activity continued for a month with smaller magnitude events forming a swarm [69,70]. Another event with similar magnitude occurred on 5 June 2007 with *M_w_* 4.8. It was shown that this seismic activity did not correlate with any of the two fault zones at the northern and southern edges of the lake, but with two unmapped NNE-SSW and NW-SE faults along its eastern shore [69,70,71]. According to [72], the existence of the water in the Trichonis Lake may have an important role, as the saturation of underlying bedrock reduces the friction coefficient, increasing the pore pressure and, consequently, decreasing the effective stress. For our analysis we used the earthquake catalogue obtained from the Seismological Laboratory of the National and Kapodistrian University of Athens (*SL-NKUA*) for the period between 8 April 2007 to 2 July 2007, counting a total of 1309 events. The minimum and maximum magnitude and depth of the catalogue is *M_L_* 1–5.2 and 0–19 km, respectively. 

#### 3.1.2. The 2016 Western Crete Earthquake Swarm

The island of Crete is located in a fore-arc position above the active northward-directed subduction zone of the African plate beneath the Aegean plate [73], where the African and Eurasian tectonic plates converge at a rate of approximately 3 cm/year [74]. The study area is located in the Southern Aegean subduction zone, which includes the outer Subduction Arc and the inner Volcanic Arc. The main fault of Western Crete is a rupture zone of significant length (>40 km) and a north–south strike, dipping to the West. Moreover, in the study area, the significant faults systems are trending N-S, also dipping westwards and composing three main segments. The oldest recorded seismic event was in 1246 with a magnitude of 6.4 [14]. The next destructive earthquakes, according to [14], took place in 1908, 1910 and 1947, with magnitudes of 6.2, 6 and 6, respectively.

The data for Western Crete were retrieved from the Institute of Physics of the Earth’s Interion and Geohazards. In this work, we studied the seismicity in Western Crete, near the village Platanos between 1 February 2016 and 25 March 2016. For the study period, the catalogue contains a total of 653 events. The strongest earthquake took place on 12 March 2016 with a magnitude of 4.8. The magnitude (*M_L_*) varies from 1 to 4.8, whereas the depth of the events is 0–26 km.

#### 3.1.3. The 2021–2022 Nisyros Earthquake Swarm

Nisyros volcano belongs to the eastern part of the South Aegean Active Volcanic Arc, between Kos and Tilos islands. It is an active volcano like the rest of the volcanoes of the Volcanic Arc, which are Methana, Sousaki, Milos and Santorini. The majority of the rocks on the island of Nisyros are Quaternary volcanic rocks represented by alternating lava flows, pyroclastic layers and viscous lava domes [75]. Several normal faults with a NE–SW strike cut through the caldera floor, with the main one being a fault striking NNW-SSE, known as Mandraki Fault [75]. In addition, the NE–SW and E–W submarine faults that have been identified in the basins surrounding Nisyros border form tectonic grabens [75]. The three strong earthquakes that took place in the area are 1493, 1490 and 412BC events, with magnitudes of 6.8, 7 and 6, respectively [14].

The earthquake catalogue was extracted from the Geodynamics Institute of the National Observatory of Athens (*GI-NOA*) to study the seismicity in the northwest area of Nisyros between 7 April 2021–30 June 2022. For this period the catalogue involves a total of 1567 events. The first of the strongest earthquakes took place on 13 April 2021 (*M_L_* 5.2), whereas on 21 June 2021 an *M_L_* 5.7 event was followed by another on 1 August 2021 with a magnitude of *M_L_* 5.4. The minimum and maximum depth of the events is 2–23 km. 

#### 3.1.4. The 2021–2022 Thiva Earthquake Swarm

Thiva is a city in Boetoia (Central Greece), located at the transition zone between the Corinth Gulf in the south and Evia in the east. Two major rift structures, oriented WNW—ESE and NW-SE, respectively, dominate the tectonics of the area [76]. The land between the two gulfs is an area of lower strain controlled by normal faulting. One of these normal fault segments is that of Kallithea. In addition, north of Thiva, a denser fault network with smaller south-dipping faults dominates the area around Yliki Lake, whereas south-dipping faults dominate in the western part of the Thiva basin and north-dipping faults in the eastern part. Historical records show that a destructive earthquake with a magnitude of 7 occurred in the Thiva region in 1853 [14] and the next large seismic event was in 1893 with a magnitude of 6 [77]. The next strongest event that is recorded in this region is an earthquake with a magnitude of *M_s_*~6.2 in 1914 [78].

In this part, we studied the seismicity in the area of Thiva between 10 July 2021 and 1 July 2022. On 2 December 2020 at 10:54:56 UTC, an earthquake with magnitude 4.5 took place east of Thiva, which was followed by a prolific seismic swarm until recently. The next strongest events were on 11 July 2021, 20 July 2021, 2 September 2021 and 10 April 2022, with magnitudes *M_w_* 4.3, 4.1, 4 and 4.4, respectively. The earthquake catalogue was obtained from the Seismological Laboratory of the National and Kapodistrian University of Athens (*SL-NKUA*). For the above period, the catalogue contains a total of 4695 events with depths from 0 to 15 km. The 2020 seismic events mainly took place at the eastern part of the study area, on a system of normal faults. On 10 July 2021, seismic activity started at the western part of the swarm and the next seismic events present a general tendency for spatiotemporal migration towards ESE. The evolution of the swarm is related to stress triggered by its major events and facilitated by pore-fluid pressure diffusion [76].

#### 3.1.5. The 2022 Pagasetic Gulf Earthquake Swarm

The Pagasetic Gulf is a semi-enclosed gulf located in the northwestern part of the Aegean Sea and is connected with North Evoikos Gulf and the Aegean Sea through the channel of Trikeri. The study area is north of the island of Evoia and southeast of the city of Volos and Ayia Kiriaki, as it is shown in Figure 1. The area is dominated by normal faulting trending NNE-WWS [79]. The main strike of faults is NE-SW, although secondary directions of NW-SE trending faults are also observed [79]. The most recent reported strong seismic event in this area is in 1753 with a magnitude of 6.3 [14].

The earthquake catalogue was extracted from the Geodynamics Institute of the National Observatory of Athens (*GI-NOA*) and consists of 283 events. This swarm lasted less than two months between 9 May 2022 and 23 June 2022, with the strongest seismic event on 11 May 2022 with a magnitude *M_L_* 3.8. The depth of the events ranges from 2 to 20 km. 

### 3.2. Frequency-Magnitude Distribution and Magnitude of Completeness

**Figure 2:**
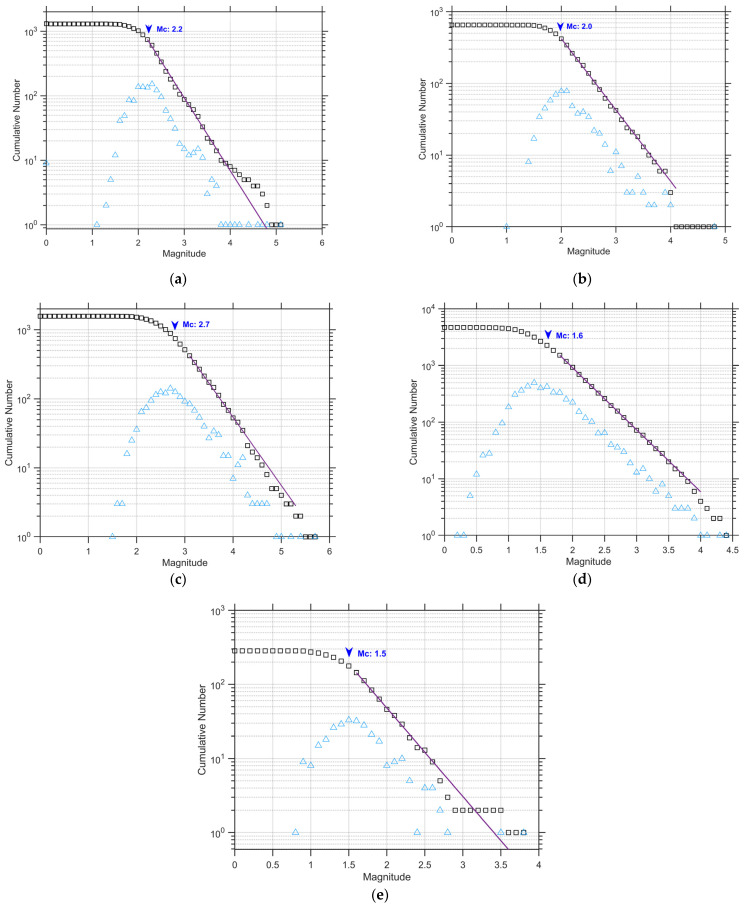
(**a**) The Gutenberg–Richter (G-R) distribution (solid line) for the 2007 Trichonis Lake Earthquake Swarm. *M_c_* is the magnitude of completeness. The frequency–magnitude distribution is represented by the cumulative (black squares) and the discrete (blue triangles) number of seismic events. The solid line represents the G-R relation. The same plots for (**b**) the 2016 Western Crete Earthquake Swarm, (**c**) the 2021–2022 Nisyros Earthquake Swarm, (**d**) the 2021–2022 Thiva Earthquake Swarm and (**e**) the 2022 Pagasetic Gulf Earthquake Swarm. The estimated values for the *a* and *b*-values, as well as for *Mc*, are given in Table 1.

In this paragraph, using the catalogues of each earthquake swarm, we estimated the magnitude of completeness (*M_c_*) using the FMD in terms of the Gutenberg–Richter scaling relation. The measurements were made by applying the Zmap software package, version 7.0, [80] which is designed for the MATLAB environment and is a useful tool in a statistical seismological analysis of earthquake datasets. According to the maximum likelihood estimation and the best combination of the maximum curvature method and the goodness-of-fit test, with 95% and 90% residuals [81], we estimated the *a* and *b* values of the Gutenberg–Richter relation and the completeness magnitude (*M_c_*) in each catalogue. The estimated values are given in Table 1. In the statistical analysis, we used only the events with *M ≥ Mc*. Figure 2 depicts the FMD for each earthquake swarm, along with the fitting according to the G-R scaling relation for the calculated model parameters.

## 4. Analysis and Results

The theory of NESP, as previously described, is herein applied to the inter-event time and distance distributions, as well as to the seismic energy distributions of the earthquake swarms. The catalogues were updated to include all earthquake events with *M* ≥ *Mc*. The inter-event time *T* is defined as *T* = *t*(*i* + 1) − *t*(*i*), where *t*(*i*) is the time of occurrence of the *i*th event, *i* = 1, 2, …, *N −* 1 and *N* is the total number of events, whereas the inter-event spatial distance is defined as the 3D Euclidean distance between the successive hypocenters. After the estimation of the cumulative inter-event time distribution *P*(>*T*), the corresponding fitting with the *Q*-exponential function, up to the value *T_c_* indicates the value *q_T_*. The parameter *T_c_* (critical inter-event time) denotes the transition points between the non-additive and additive behavior. In most of the cases that we study, we observe the deviation from the *Q*-exponential function for high values of *T*, with *T* > *T_c_*. The crossover points between the non-additive and additive behavior are indicated by the deviation from linearity at *T* > *T_c_* in the *Q*-logarithmic functions ln*_Q_P*(>*T*). Following the same procedure, we estimate the cumulative interevent distances distribution *P*(>*D*) that provides the *q_D_* parameter value. After the calculation of the appropriate q that describes the observed distributions *P*(>*T*) and *P*(>*D*), the *Q*-logarithmic functions ln*_Q_P*(>*T*) and ln*_Q_P*(>*D*) are linear with *T* (*T* < *T_c_*) and *D*, respectively.

**Figure 3:**
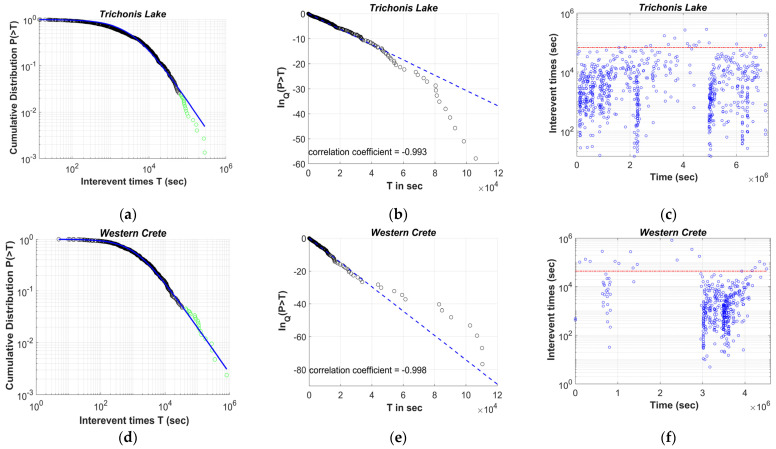

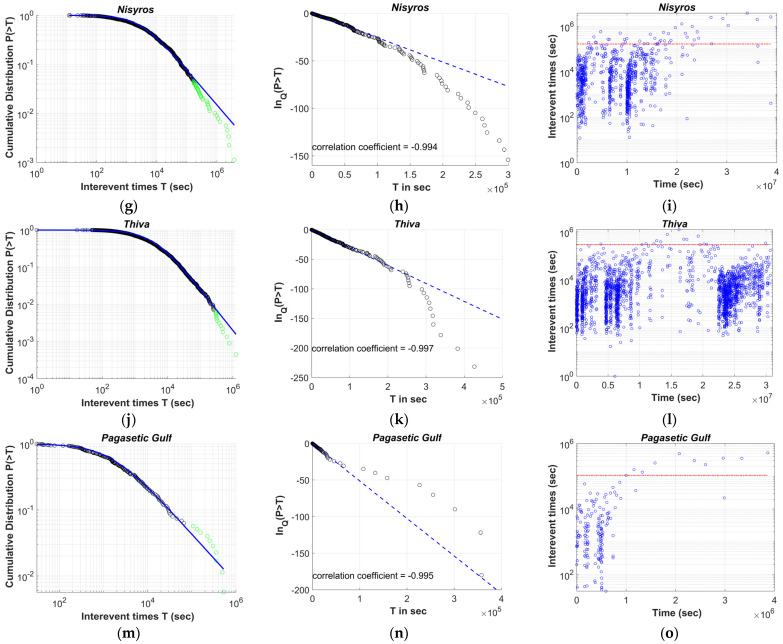
**Left panels** (**a**,**d**,**g**,**j**,**m**) The log-log plot of the inter-event times cumulative distribution for *M* ≥ *Mc*, represented by circles. The change of colors indicates the transition from NESP (black circles) to Boltzmann–Gibbs regimes (green circles). The blue solid line represents the *Q*-exponential distribution fitting, for the values of (**a**) *q_T_* = 1.44, *T_q_* = 1805 s, (**d**) *q_T_* = 1.53, *T_q_* = 633 s, (**g**) *q_T_* = 1.58, *T_q_* = 1625 s, (**j**) *q_T_* = 1.47, *T_q_* = 1736 s, (**m**) *q_T_* = 1.57, *T_q_* = 829 s. **Middle panels** (**b**,**e**,**h**,**k**,**n**) The corresponding semi-*q*-log plot of the cumulative distribution of the inter-event times (*T*), represented by circles. The *Q*-logarithmic distribution exhibits a correlation coefficient (*ρ*) and the dashed line is the *Q*-exponential fitting with q_T_. **Right panels** (**c**,**f**,**i**,**l**,**o**) The evolution of inter-event times (*T*) with time (*t*). The deviation from linearity suggests a *T_c_* value, presented in Table 2, which is indicated by the red dashed line and shows the crossover point between NESP and BG statistical physics.

The results of the analysis are presented in Figure 3, in terms of the cumulative distribution *P*(>*T*) of the inter-event times *T* for each earthquake swarm. The *Q*-exponential fitting uses the generalized expression of entropy, Equation (1) describes the observed distributions quite well, leading to the parameters *q_T_* and *T_q_* (Table 2). The transition from NESP (black circles) to Boltzmann–Gibbs (green circles) scaling regimes is indicated by the color change in Figure 3. The corresponding *Q*-logarithmic distribution ln*_Q_*(*P*(>*T*)) for each earthquake swarm is shown in the middle panels of Figure 3, while the evolution of inter-event times (*T*) as a function of time (*t*) is shown in the right panels of Figure 3. The red dashed line (*T_c_* value) shows that most inter-event times have *T* values less than *T_c_*, indicating that the Tsallis entropic mechanism is predominant in the main part of the swarm. However, as time passes the characteristics of the earthquake swarm such as those of finite degree of freedom and long-range memory, related to a non-extensive statistical physics description are not any more predominant and the Boltzmann-Gibbs statistical physics is recovered (i.e., *q* = 1).

In particular, for the 2007 Trichonis Lake swarm, the *Q*-exponential distribution describes well the observed scaling behavior for the values of *q_T_* = 1.44 and *T_q_* = 1805 s and up to a characteristic time *T_c_* = 6.8 × 10^4^ s, where a fall-off in the distribution appears, probably due to the finite size of the swarm, limiting the occurrence of sporadic events and hence the emergence of long inter-event times (finite-size effect). Similarly, for the 2016 Western Crete and the 2021*–*2022 Nisyros swarms, the *Q*-exponential distribution describes quite well the observed *P*(>*T*) for the values of *q_T_* = 1.53, *T_q_* = 633 s, *T_c_* =4.3 × 10^4^ s and *q_T_* = 1.58, *T_q_* = 1625 s, *T_c_* = 1.7 × 10^5^ s, respectively. In addition, for the 2021*–*2022 Thiva and the 2022 Pagasetic Gulf swarms, the *Q*-exponential fitting using Equation (7) describes the observed data well, leading to *q_T_* = 1.47, *T_q_* = 1736 s, *T_c_* = 2.9 × 10^5^ sec and *q_T_* = 1.57, *T_q_* = 829 s, *T_c_* = 1.1 × 10^5^ s, respectively. The higher than unity *q_T_*-values indicate asymptotic power-law behavior and long-range correlations in the temporal evolution of the earthquake activity [26]. The corresponding *Q*-logarithmic distributions for each earthquake swarm, describe the observed distributions with high correlation coefficients, as shown in the middle panels (Figure 3), supporting the goodness-of-fit between the model and the data. 

**Figure 4:**
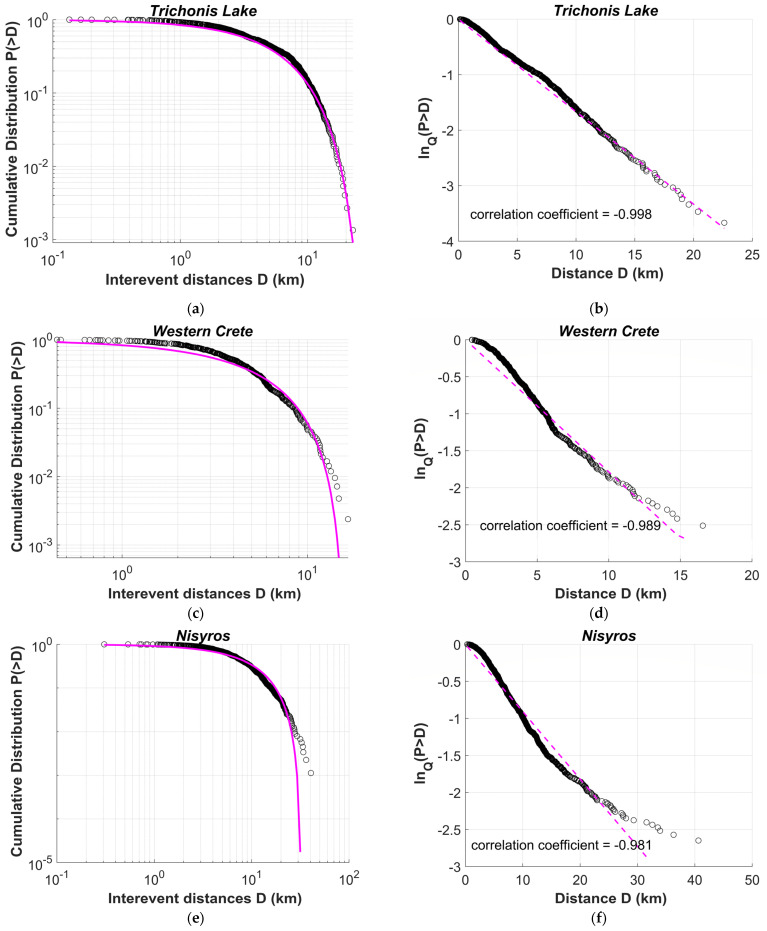

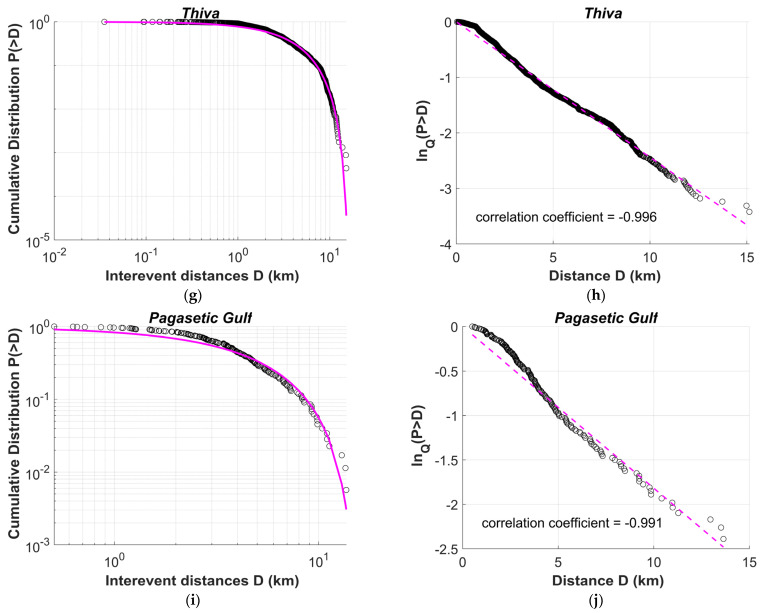
**Left panels** (**a**,**c**,**e**,**g**,**i**) The log-log plot of the inter-event distances cumulative distribution for *M* ≥ *M_c_*, represented by black circles. The magenta solid line represents the *Q*-exponential distribution fitting for the values of (**a**) *q_D_* = 0.75, *D_q_* = 7.5 km, (**c**) *q_D_* = 0.46, *D_q_* = 8.62 km, (**e**) *q_D_* = 0.48, *D_q_* = 16.67 km, (**g**) *q_D_* = 0.67, *D_q_* = 5.74 km, (**i**) *q_D_* = 0.76, *D_q_* = 8.46 km. **Right panels** (**b**,**d**,**f**,**h**,**j**) The corresponding semi-*q*-log plot of the cumulative distribution of the inter-event distances (*D*), indicated by black circles. The *Q*-logarithmic distribution exhibits a correlation coefficient of (*ρ*) and the solid line is the *Q*-exponential fitting with q_D_.

In Figure 4, the cumulative distributions *P*(>*D*) of inter-event distances *D* are presented, showing a good fit with the *Q*-exponential function for *q_D_* < 1. The q and *D_q_* values estimated from the analysis are presented in Table 2 for each earthquake swarm. The corresponding *Q*-logarithmic distributions ln*_Q_*(*P*(>*D*)) are also plotted in the middle panel of Figure 4, with the straight dashed line indicating the *Q*-exponential function that approaches linearity with a *ρ* correlation coefficient. The estimated *q_T_* or *q_D_* parameters give the best linear fitting. 

The *Q*-exponential distributions for the 2007 Trichonis Lake, the 2016 Western Crete and the 2021*–*2022 Nisyros swarms, describe well the observed scaling behavior for the values of *q_D_* = 0.75, *D_q_* = 7.5 km and *q_D_* = 0.46, *D_q_* = 8.62 km and *q_D_* = 0.48, *D_q_* = 16.67 km, respectively. Furthermore, for the 2021*–*2022 Thiva and the 2022 Pagasetic Gulf swarms, the *Q*-exponential distributions describe well the observed *P*(>*D*) for the values of *q_D_* = 0.67, *D_q_* = 5.47 km and *q_D_* = 0.46, *D_q_* = 8.46 km. The interevent distances of the seismic activity deviate from the exponential function, indicating organization rather than random spatial occurrence [26]. The corresponding *Q*-logarithmic distributions approximate linearity with high correlation coefficients, indicating the goodness-of-fit between the model and the data. According to our results, the cumulative distribution functions of inter-event times and distances are quite well described with the *Q*-exponential function, with an entropic parameter *q* greater than one (*q* > 1) for inter-event times and less than one (*q* < 1) for the inter-event distances, further confirming the results of [35,47,49,82,83].

### The Frequency–Magnitude Distribution and the Fragment–Asperity Model

**Figure 5:**
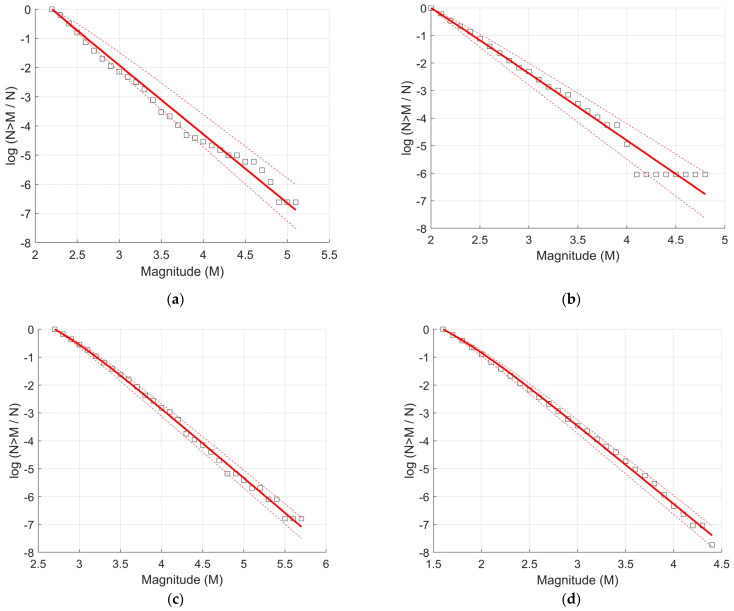

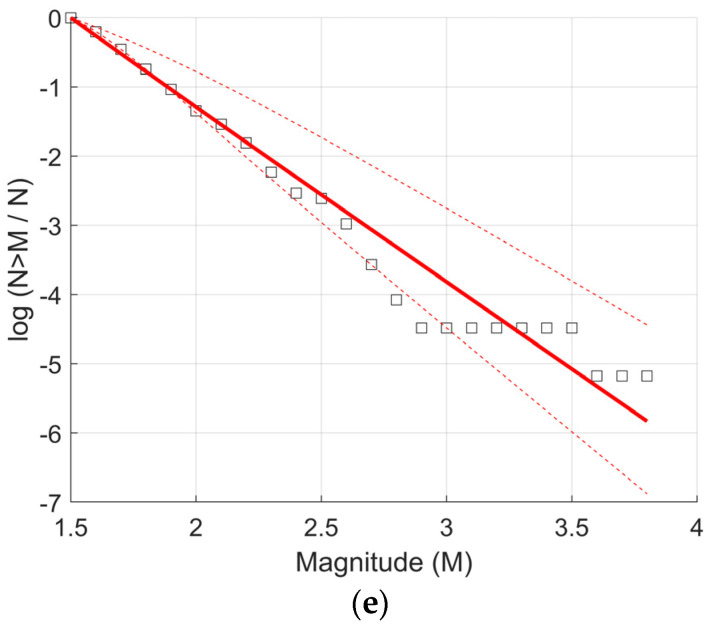
(**a**) Normalized cumulative magnitude distribution of the 2007 Trichonis Lake Earthquake Swarm (squares) and the fitting (solid line) according to the fragment–asperity interaction model (Equation (10)) for the value of *q_M_* = 1.48. The 95% confidence intervals are plotted with dashed lines. (**b**) The same plot for the 2016 Western Crete Earthquake Swarm, (**c**) the 2021*–*2022 Nisyros Earthquake Swarm, (**d**) the 2021*–*2022 Thiva Earthquake Swarm, (**e**) the 2022 Pagasetic Gulf Earthquake Swarm. The estimated values for each *q_M_* along with the 95% confidence intervals are given in Table 3.

In this paragraph, we have applied the NESP model of Equation (10) to the normalized cumulative magnitude distribution of each earthquake swarm (Figure 5), for the entire magnitude range above a threshold that equals the magnitude of completeness for each earthquake swarm (Table 1). The model describes rather well the observed magnitude distribution for the 2007 Trichonis Lake, the 2016 Western Crete, the 2021*–*2022 Nisyros, the 2021*–*2022 Thiva and the 2022 Pagasetic Gulf earthquake swarms, while the fitting of Equation (10) to the observed data leads to *q_M_* = 1.49, *q_M_* = 1.48, *q_M_* = 1.48, *q_M_* = 1.45, *q_M_* = 1.48, respectively (Table 3). In Figure 5, the bold red line shows the model of Equation (10) for the estimated non-extensive parameter *q_M_*, while the other two dashed lines represent the 95% confidence intervals (Table 3). The above analysis indicates that the fragment–asperity model describes quite well the seismic behavior. The high correlation between the model of Equation (10) and the observed seismicity is an indication of the NESP model’s applicability and success in identifying the major characteristics of earthquake dynamics.

The distributions reflect sub-extensive systems, where long-range interactions are significant [61,84]. The analysis of the cumulative earthquake distribution gives *q_M_* values between 1.45–1.49 for the studied earthquake swarms. These results suggest that the areas are away from equilibrium in a statistical physics sense, which means that the fault planes and fragments filling the gap between them are not in equilibrium, leading to an increased seismic activity to be expected [85]. The decrease in the non-extensivity parameter *q_M_* are observed when small-magnitude earthquakes occur, as in the case of Thiva (*q_M_* = 1.45). This could reveal that the order within the system of faults is decreasing, and the amount of accumulated stress is not yet enough to initiate a correlated behavior of the whole system [86]. High values of q_M_ are found in the regions where large earthquakes have occurred, which is in agreement with previous results [30,31,51,62,82,84,86,87]. When a strong earthquake occurs and much more correlated behavior of the system constituents is assumed to take place, short- and long-range magnitude correlations emerge, inducing an increase in the non-extensivity parameter *q_M_* [87]. Trichonis Lake is an active seismic area in which many strong earthquakes have taken place in the past and that explains the high value of *q_M_* = 1.49. 

## 5. Discussion

The concept of Non-Extensive Statistical Physics (NESP) is herein applied to the inter-event time and distance distributions of recent earthquake swarms that have recently occurred in Greece. We observed that the cumulative distribution functions of the inter-event times and distances between the successive earthquakes for all the studied swarm sequences are well described by the *Q*-exponential function (Equation (1)). In the case of inter-event times, a deviation from the *Q*-exponential function appears for high values of *T* (*T > T_c_*), where *T_c_* indicates the transition between the non-additive and additive behavior. For each earthquake swarm, we estimated the entropic parameter *Q* by fitting the *Q*-exponential function to the observed data up to a value close to *T_c_* and then we calculated the parameter *q* from the equation $q=2-\left(1/Q\right)$. According to Abe and Suzuki [34,35], the three-dimensional distances of successive earthquakes expressed by the *Q*-exponential distribution and the corresponding *q_D_* values are less than unity *q_D_* < 1, whereas the cumulative distribution of the inter-event times is described by the *Q*-exponential function with *q_T_* > 1. They also proposed the relation: $q_{T}+q_{D}\approx 2$ which is observed in seismicity data from California and Japan [34,35] and Iran [88], and verified numerically using the two-dimensional Burridge–Knopoff model [89,90]. Furthermore, this value is in agreement with previous studies [41,83,91]. Our results further confirm these patterns for recent earthquake swarms in Greece, indicating that the cumulative probability distribution of the inter-event times *P*(*>T*) and inter-event distances *P*(*>D*) are adequately described with the *Q*-exponential function, with *q_T_ > 1* and *q_D_ < 1*, respectively. The latter indicates a correlated process in time and space that deviates from the random case and the exponential distribution. In addition, the inter-event times exhibit an asymptotic power-law behavior, whereas for inter-event distances a cut-off appears. In this last case, a cut-off in the inter-event distances distribution seems to be the appropriate scaling behavior for real data [92] due to the finite size effects of the seismogenic crust [18]. This scaling behavior with *q* < 1 has been verified in previous earthquake studies [26,35,83,91]. More specifically, the estimated non-extensive *q*-values that characterized the observed inter-event time distributions are within the range of 1.44–1.58, whereas for inter-event distances are from 0.46 to 0.75. The entropic parameters *q_T_***,** *q_D_***,** *T_q_* and *D_q_* along with their 95% confidence intervals and the critical parameter *Tc*, are summarized in Table 2. Furthermore, in Table 4, we point out that the sum of *q_T_* and *q_D_* parameters of the inter-event time and distance distributions for each earthquake swarm is approximately $q_{T}+q_{D}\approx 2$.

The cumulative inter-event time and distance distribution functions for each earthquake swarm are well described with the *Q*-exponential function, indicating asymptotic power–law behavior and long-term correlations in the spatiotemporal evolution of seismicity. In addition, the *q*-values of *q_T_* > 1 and *q_D_* < 1 are in agreement with the *q*-values found for aftershock sequences [38,40,83,93,94], for the Hellenic Subduction Zone [91], for the temporal properties of seismicity [60,61] and for global seismicity [41,84]. In addition, the concept of NESP describes well both the spatial and temporal behavior of the earthquake swarms in diverse tectonic environments [7,18,26,95] and in volcanic regions [23,59]. Moreover, the value of *q_T_* > 1 suggests a sub-additive process, leading to the conclusion of long-range memory in the evolution of earthquake swarms for *T < T_c_.* The framework of NESP that was used in the present study describes well the spatiotemporal scaling properties of the earthquake swarms, as well as the seismic energy distributions. Hence, the application of non-extensive statistical physics represents a quite useful tool in investigating such phenomena, exhibiting scale-free nature and long-range memory effects. 

The observed deviation from the *Q*-exponential function for high *T* values could be explained by introducing two mechanisms that drive the earthquake swarms. The first one is governed by Tsallis entropy and is dominant for inter-event times with *T < T_c_*, whereas the second is observed for *T > T_c_* and is defined by an exponential function (i.e., *q* = 1 in NESP terms). Thus, in order to include a crossover from non-additive (*q* ≠ 0) to additive (*q* = 1) behavior, we apply a generalization of anomalous equilibrium distributions described in [96,97], where:

(12) $$\frac{dp}{dT}=-\beta_{1}p-\left(\beta_{q}-\beta_{1}\right)p^{q}$$

whose solution is:

(13) pT=C 1−βqβ1+βqβ1eq−1β1T11−q

where *C* is a normalization factor and for positive *β_q_* and *β*_1_, *p*(*T*) decreases monotonically with increasing *T*. In the case where $\left(q-1\right)\beta_{1}\ll 1$, Equation (13) can be approximated as *q*-exponential, $p\left(T\right)\approx C\exp_{q}(-T/T_{q})$, where $T_{q}=1/\beta_{q}$ while for $\left(q-1\right)\beta_{1}\ll 1$, the asymptotic behavior of the probability distribution is $p\left(T\right)\propto {\left(\frac{\beta_{1}}{\beta_{q}}\right)}^{1/\left(q-1\right)}e^{-\beta_{1}T}$, an exponential function, where $T_{c}=1/\left(q-1\right)\beta_{1}$ is the crossover point between non-additive and additive behavior [46].

Many complex systems exhibit inhomogeneous spatiotemporal dynamics that can be characterized by a superposition of numerous generalized statistics on different scales, called ’superstatistics’, which is complementary to NESP [42,43,44]. Superstatistics is a non-extensive statistical physics approach to understanding its dynamic reason [44]. The *Q*-exponential behavior of the inter-event times can be observed in terms of superstatistics, which are based on a superposition of ordinary local equilibrium states, controlled by an intensive parameter (*β*) that fluctuates on a relatively large spatiotemporal scale and is supplementary to NESP [39,42,43,44]. For a superstatistical approach to all the above earthquake swarms, a very simple model, where the local distributions are expressed by that of a Poisson process p(*T*|*β*)$=\beta e^{-\beta Τ}$, is assumed, with p(*T*|*β*) expressing the conditional probability density of the inter-event times given that it has an average value *T*|*β* and the intensive parameter β that fluctuates on a relatively large scale, leading the exponential model to become superstatistical. If the parameter *β* is distributed with probability density *f*(*β*)**,** then the probability distribution is given as:

(14) $$p\left(T\right)=\int_{0}^{\infty }f\left(\beta \right)p\left(T|\beta \right)d\beta =\int_{0}^{\infty }f\left(\beta \right)\beta e^{-\beta Τ}d\beta$$

There can be *n* Gaussian random variables *X*_1_, ..., *Xn* of the same variance due to various relevant degrees of freedom in the system [43]. The simplest way to get a positive *β* is to square the Gaussian random variables and sum them up. Hence, as a result $\beta =\overset{n}{\underset{i=0}{\sum }}X_{i}^{2}$ where $X_{i}^{2}\neq 0,\;X_{i}\neq 0$.

The probability density of *β* is *X*^2^-distributed with *n* degrees of freedom:

(15) $$f\left(\beta \right)=\frac{1}{\Gamma \left(n/2\right)}{\left(\frac{n}{2\beta_{0}}\right)}^{n/2}\;\;\beta^{n/2-1}\exp\left(-\frac{n\beta }{2\beta_{0}}\right)$$

where *β*_0_ is the average of *β*.

The integration in Equation (14) using Equation (15) has as a result the generalized canonical distribution of NESP:

(16) $$p\left(T\right)\approx C{\left(1+B\left(q-1\right)T\right)}^{1/\left(1-q\right)}$$

where *C* is a normalization factor and $q=1+2/\left(n+2\right)$, $B=2\beta_{0}/\left(2-q\right)$.

The calculation of the *n* degrees of freedom that are influencing the value of *β*, can be performed according to [38]:

(17) $$n=\frac{2}{q-1}-2\;$$

Using the *q_T_* value of each earthquake swarm (Table 2), acquired from our *q*-statistics fits, we can estimate the *n* degrees of freedom for each earthquake swarm (Table 4). The degrees of freedom obtained from Equation (17) are non-integer values and hence, we report the nearest integer as a result. The low number of *n*, ranging between 1 and 3, indicates the spatiotemporal organization in the evolution of the swarm activity. The high degrees of freedom imply the loss of temporal correlations and close proximity to Poissonian (random) behavior, such as in the case of Trichonis Lake with *n* = 3, whereas the low degrees of freedom deviate from the random case and indicate a correlated process in time and space.

## 6. Conclusions

In the present work, the spatiotemporal scaling properties and the frequency-magnitude distribution of earthquake swarms that have recently occurred within the area of Greece were investigated using the framework of Non-Extensive Statistical Physics (NESP). The analysis and results indicate power-law asymptotic behavior in the inter-event time distributions that scale according to the *Q*-exponential function up to a characteristic inter-event time *T_c_*. The range of the *q_T_* parameter is found between 1.44 and 1.58, supporting the idea of the presence of long-range memory effects in the evolution of seismicity. In addition, the inter-event distances distributions are described with the Q-exponential function with *q_D_*-values below unity and in the range of 0.46*–*0.75, leading to a cut-off in the tail of the distribution. The sum of these parameters that describe the inter-event times, *q_T_* > 1, and distances, *q_D_* < 1, distributions, is $q_{T}+q_{D}\approx 2$. In addition, we use the Gutenberg–Richter scaling relation and the fragment–asperity model to describe the frequency–magnitude distributions of the earthquake swarms. The frequency of swarm magnitudes follows the Gutenberg–Richter relation for b-values varying between 0.99 and 1.19. Moreover, the fragment–asperity model, with values of the entropic index *q_M_* in the range of 1.45 to 1.49, reproduces the complexity in the earthquake energy distribution quite effectively. Lastly, we applied a superstatistic approach, which is based on a superposition of ordinary local equilibrium statistical mechanics with an appropriate intensive parameter *β* that fluctuates as *χ*^2^ distribution on a rather large temporal scale. The superstatistical approach leads to the conclusion that in each earthquake swarm, the temporal evolution is described by low degrees of freedom, indicating a high level of organization, hierarchy and non-additive characteristics. 

## Figures and Tables

**Table 1:** *N* the number of earthquake events, *Mc* the completeness magnitude, *Nc* the number of the events with *M ≥ Mc*, *a* and *b* the values in the Gutenberg–Richter distribution, for each earthquake swarm.

Swarms	*N*	*Mc*	*Nc*	*b*	*a*
Trichonis Lake	1309	2.2	745	1.13 ± 0.04	5.36
Western Crete	653	2.0	420	0.99 ± 0.05	4.61
Nisyros	1567	2.7	887	0.99 ± 0.05	5.69
Thiva	4695	1.6	2275	1.10 ± 0.03	5.15
Pagasetic Gulf	283	1.5	177	1.19 ± 0.1	4.07

**Table 2:** The non-extensive parameters *q_T_*, *T*_0_, *q_D_* and *D*_0_ introduced in NESP to describe the spatiotemporal evolution of earthquake swarms, along with their 95% confidence intervals. *q_T_*, *q_D_* represent the entropic index, *T_q_*, *D_q_* are the generalized scaled interevent time–distance and *T_c_* is the critical interevent time.

Swarms	*q_T_*	*T_q_* (s)	*T_q_*_1_–*T_q_*_2_	*q_D_*	*D_q_* (km)	*D_q_*_1_–*D_q_*_2_	*Τ_c_* (s)
Trichonis Lake	1.44	1805	1781–1829	0.75	7.5	7.43–7.57	6.8 × 10^4^
Western Crete	1.53	633	629–638	0.46	8.62	8.40–8.83	4.3 × 10^4^
Nisyros	1.58	1625	1610–1640	0.48	16.67	16.45–16.88	1.7 × 10^5^
Thiva	1.47	1736	1731–1742	0.67	5.47	5.40–5.52	2.9 × 10^5^
Pagasetic Gulf	1.57	829	821–837	0.46	8.46	8.15–8.70	1.1 × 10^5^

**Table 3:** *q_M_* is the entropic index for each earthquake swarm with 95% confidence intervals.

Swarms	*q_M_*	*q_M_*_1_–*q_M_*_2_
Trichonis Lake	1.49	1.47–1.51
Western Crete	1.48	1.46–1.50
Nisyros	1.48	1.47–1.49
Thiva	1.45	1.44–1.46
Pagasetic Gulf	1.48	1.44–1.52

**Table 4:** The parameter *q_T_* + *q_D_*, along with the number *n* of the degrees of freedom, as estimated from the superstatistical model. As degrees of freedom, we select the nearest integer to Equation (17).

Swarms	*q_T_* + *q_D_*	*n*
Trichonis Lake	2.19	3
Western Crete	1.99	2
Nisyros	2.06	1
Thiva	2.14	2
Pagasetic Gulf	2.03	2

## Data Availability

Data are openly available at the Seismological Laboratory of the National and Kapodistrian University of Athens (SL-NKUA, http://www.geophysics.geol.uoa.gr/) for Thiva (Last accessed on 2 July 2022) and at the Geodynamics Institute of National Observatory of Athens (GI-NOA, http://bbnet.gein.noa.gr/) for Nisyros and Pagasetic Gulf. (Last accessed on 2 July 2022). The data for Western Crete are taken from [98] (Institute of Physics of the Earth’s Interion and Geohazards (https://earth-phys.hmu.gr/, last accessed on 2 July 2022)) and are available upon request from (G.C.) The data for Trichonis Lake are taken from [72] (SL-NKUA) and are available upon request from (A.K.).

## References

[1] Mogi K. (1963). Some Discussions on Aftershocks, Foreshocks and Earthquake Swarms: The Fracture of a Semi-Infinite Body Caused by an Inner Stress Origin and Its Relation to the Earthquake Phenomena.

[2] Špičák A. (2000). Earthquake swarms and accompanying phenomena in intraplate regions: A Review. Stud. Geophys. Geod..

[3] Vidale J.E., Shearer P. (2006). A survey of 71 earthquake bursts across southern California: Exploring the role of pore fluid pressure fluctuations and aseismic slip as drivers. J. Geophys. Res. Solid Earth.

[4] Chen X., Shearer P.M., Abercrombie R.E. (2012). Spatial migration of earthquakes within seismic clusters in Southern California: Evidence for fluid diffusion. J. Geophys. Res. Solid Earth.

[5] Bhattacharya S.N., Dattatrayam R.S. (2003). Some Characteristics of recent earthquake sequences. Gondwana Geol. Mag..

[6] Hainzl S., Kraft T., Wassermann J., Igel H., Schmedes E. (2006). Evidence for rainfall-triggered earthquake activity. Geophys. Res. Lett..

[7] Michas G., Kapetanidis V., Spingos I., Kaviris G., Vallianatos F. (2022). The 2020 Perachora peninsula earthquake sequence (Εast Corinth Rift, Greece): Spatiotemporal evolution and implications for the triggering mechanism. Acta Geophys..

[8] Kundu B., Legrand D., Gahalaut K., Gahalaut V.K., Mahesh P., Raju K.A.K., Catherine J.K., Ambikapthy A., Chadha R.K. (2012). The 2005 volcano-tectonic earthquake swarm in the Andaman Sea: Triggered by the 2004 great Sumatra-Andaman earthquake: SWARM IN THE ANDAMAN SEA. Tectonics.

[9] Sateesh A., Mahesh P., Singh A.P., Kumar S., Chopra S., Kumar M.R. (2019). Are earthquake swarms in South Gujarat, northwestern Deccan Volcanic Province of India monsoon induced?. Environ. Earth Sci..

[10] Srijayanthi G., Chatterjee R., Kamra C., Chauhan M., Chopra S., Kumar S., Chauhan P., Limbachiya H., Ray P.C. (2022). Seismological and InSAR based investigations to characterise earthquake swarms in Jamnagar, Gujarat, India—An active intraplate region. J. Asian Earth Sci. X.

[11] Makropoulos K., Burton P.W. (1984). Greek tectonics and seismicity. Tectonophysics.

[12] Tsapanos T.M., Burton P.W. (1991). Seismic hazard evaluation for specific seismic regions of the world. Tectonophysics.

[13] Kouskouna V., Makropoulos K. (2009). Historical earthquake investigations in Greece. Ann. Geophys..

[14] Papazachos B.C., Papazachou C. (2003). The Earthquakes of Greece.

[15] Tselentis G.-A., Stavrakakis G., Makropoulos K., Latousakis J., Drakopoulos J. (1988). Seismic moments of earthquakes at the western Hellenic arc and their application to the seismic hazard of the area. Tectonophysics.

[16] Varotsos P., Sarlis N., Skordas E., Lazaridou M. (2006). Additional evidence on some relationship between Seismic Electric Signals (SES) and earthquake focal mechanism. Tectonophysics.

[17] Pacchiani F., Lyon-Caen H. (2010). Geometry and spatio-temporal evolution of the 2001 Agios Ioanis earthquake swarm (Corinth Rift, Greece). Geophys. J. Int..

[18] Michas G., Vallianatos F. (2018). Modelling earthquake diffusion as a continuous-time random walk with fractional kinetics: The case of the 2001 Agios Ioannis earthquake swarm (Corinth Rift). Geophys. J. Int..

[19] Duverger C., Godano M., Bernard P., Lyon-Caen H., Lambotte S. (2015). The 2003–2004 seismic swarm in the western Corinth rift: Evidence for a multiscale pore pressure diffusion process along a permeable fault system. Geophys. Res. Lett..

[20] Kapetanidis V., Deschamps A., Papadimitriou P., Matrullo E., Karakonstantis A., Bozionelos G., Kaviris G., Serpetsidaki A., Lyon-Caen H., Voulgaris N. (2015). The 2013 earthquake swarm in Helike, Greece: Seismic activity at the root of old normal faults. Geophys. J. Int..

[21] Mesimeri M., Karakostas V., Papadimitriou E., Schaff D., Tsaklidis G. (2016). Spatio-temporal properties and evolution of the 2013 Aigion earthquake swarm (Corinth Gulf, Greece). J. Seism..

[22] De Barros L., Cappa F., Deschamps A., Dublanchet P. (2020). Imbricated Aseismic Slip and Fluid Diffusion Drive a Seismic Swarm in the Corinth Gulf, Greece. Geophys. Res. Lett..

[23] Vallianatos F., Michas G., Papadakis G., Tzanis A. (2013). Evidence of non-extensivity in the seismicity observed during the 2011–2012 unrest at the Santorini volcanic complex, Greece. Nat. Hazards Earth Syst. Sci..

[24] Saltogianni V., Stiros S.C., Newman A.V., Flanagan K., Moschas F. (2014). Time-space modeling of the dynamics of Santorini volcano (Greece) during the 2011-2012 unrest. J. Geophys. Res. Solid Earth.

[25] Mesimeri M., Karakostas V., Papadimitriou E., Tsaklidis G., Tsapanos T. (2017). Detailed microseismicity study in the area of Florina (Greece): Evidence for fluid driven seismicity. Tectonophysics.

[26] Michas G., Vallianatos F. (2020). Scaling properties and anomalous diffusion of the Florina micro-seismic activity: Fluid driven?. Géoméch. Energy Environ..

[27] Kostoglou A., Karakostas V., Bountzis P., Papadimitriou E. (2020). Τhe February–March 2019 Seismic Swarm Offshore North Lefkada Island, Greece: Microseismicity Analysis and Geodynamic Implications. Appl. Sci..

[28] Gutenberg B., Richter C.F. (1944). Frequency of earthquakes in California. Bull. Seism. Soc. Am..

[29] Sotolongo-Costa O., Posadas A. (2004). Fragment-Asperity Interaction Model for Earthquakes. Phys. Rev. Lett..

[30] Motaghed S., Khazaee M., Eftekhari N., Mohammadi M. (2023). A non-extensive approach to probabilistic seismic hazard analysis. Nat. Hazards Earth Syst. Sci..

[31] Vega-Jorquera P., de la Barra E., da Silva S.L.E. (2023). Antropogenic seismicity and the breakdown of the self-similarity described by nonextensive models. Phys. A Stat. Mech. Appl..

[32] Tsallis C., Abe S., Okamoto Y. (2001). Nonextensive Statistical Mechanics and Thermodynamics: Historical Backgroundand Present Status.

[33] Vallianatos F., Benson P., Meredith P., Sammonds P. (2012). Experimental evidence of a non-extensive statistical physics behaviour of fracture in triaxially deformed Etna basalt using acoustic emissions. EPL.

[34] Abe S., Suzuki N. (2005). Scale-free statistics of time interval between successive earthquakes. Phys. A Stat. Mech. Appl..

[35] Abe S., Suzuki N. (2003). Law for the distance between successive earthquakes. J. Geophys. Res. Solid Earth.

[36] Sarlis N., Skordas E., Varotsos P. (2010). Nonextensivity and natural time: The case of seismicity. Phys. Rev. E.

[37] Abe S., Suzuki N. (2004). Aging and scaling of aftershocks. Phys. A.

[38] Vallianatos F., Karakostas V., Papadimitriou E. (2014). A Non-Extensive Statistical Physics View in the Spatiotemporal Properties of the 2003 (Mw6.2) Lefkada, Ionian Island Greece, Aftershock Sequence. Pure Appl. Geophys..

[39] Vallianatos F., Pavlou K. (2021). Scaling properties of the Mw7.0 Samos (Greece), 2020 aftershock sequence. Acta Geophys..

[40] Rotondi R., Bressan G., Varini E. (2022). Analysis of temporal variations of seismicity through non-extensive statistical physics. Geophys. J. Int..

[41] Vallianatos F., Sammonds P. (2013). Evidence of non-extensive statistical physics of the lithospheric instability approaching the 2004 Sumatran–Andaman and 2011 Honshu mega-earthquakes. Tectonophysics.

[42] Beck C., Cohen E. (2003). Superstatistics. Phys. A Stat. Mech. Appl..

[43] Beck C. (2009). Recent developments in superstatistics. Braz. J. Phys..

[44] Beck C. (2001). Dynamical Foundations of Nonextensive Statistical Mechanics. Phys. Rev. Lett..

[45] Tsallis C. (1988). Possible generalization of Boltzmann-Gibbs statistics. J. Stat. Phys..

[46] Tsallis C. (2009). Introduction to Nonextensive Statistical Mechanics.

[47] Vallianatos F., Michas G., Papadakis G., D’Amico S. (2016). A description of seismicity based on non-extensive statistical physics: A review. Earthquakes and Their Impact on Society.

[48] Livadiotis G., McComas D.J. (2022). Physical Correlations Lead to Kappa Distributions. Astrophys. J..

[49] Vallianatos F., Papadakis G., Michas G. (2016). Generalized statistical mechanics approaches to earthquakes and tectonics. Proc. R. Soc. A: Math. Phys. Eng. Sci..

[50] Picoli S., Mendes R.S., Malacarne L.C., Santos R.P.B. (2009). q-distributions in complex systems: A brief review. Braz. J. Phys..

[51] Sigalotti L.D.G., Ramírez-Rojas A., Vargas C.A. (2023). Tsallis q-Statistics in Seismology. Entropy.

[52] Ferri G.L., Martínez S., Plastino A. (2005). Equivalence of the four versions of Tsallis’s statistics. J. Stat. Mech. Theory Exp..

[53] Wada T., Scarfone A. (2005). Connections between Tsallis’ formalisms employing the standard linear average energy and ones employing the normalized q-average energy. Phys. Lett. A.

[54] Vallianatos P., Sammonds (2011). A non-extensive statistics of the fault-population at the Valles Marineris extensional prov-ince. Mars. Tectonophys..

[55] Frohlich C., Davis S.D. (1993). Teleseismic *b* values; Or, much ado about 1.0. J. Geophys. Res. Atmos..

[56] Varotsos P.A., Sarlis N.V., Skordas E.S., Tanaka H. (2004). A plausible explanation of the b-value in the Gutenberg-Richter law from first Principles. Proc. Jpn. Acad. Ser. B.

[57] Scholz C.H. (2019). The Mechanics of Earthquakes and Faulting.

[58] McNutt S.R. (1996). Seismic Monitoring and Eruption Forecasting of Volcanoes: A Review of the State-of-the-Art and Case Histories. Moni-Toring and Mitigation of Volcano Hazards.

[59] Chochlaki K., Michas G., Vallianatos F. (2018). Complexity of the Yellowstone Park Volcanic Field Seismicity in Terms of Tsallis Entropy. Entropy.

[60] Antonopoulos C.G., Michas G., Vallianatos F., Bountis T. (2014). Evidence of q-exponential statistics in Greek seismicity. Phys. A Stat. Mech. Appl..

[61] Michas G., Vallianatos F., Sammonds P. (2013). Non-extensivity and long-range correlations in the earthquake activity at the West Corinth rift (Greece). Nonlinear Process. Geophys..

[62] Telesca L. (2011). Tsallis-Based Nonextensive Analysis of the Southern California Seismicity. Entropy.

[63] Telesca L. (2012). Maximum Likelihood Estimation of the Nonextensive Parameters of the Earthquake Cumulative Magnitude Distribution. Bull. Seism. Soc. Am..

[64] Papadakis G., Vallianatos F., Sammonds P. (2015). A Nonextensive Statistical Physics Analysis of the 1995 Kobe, Japan Earthquake. Pure Appl. Geophys..

[65] Aki K. (1965). Maximum likelihood estimate of b in the formula logN = a − bM and its confidence limits. Bull. Earthq. Res. Inst. Tokyo Univ..

[66] Michas G. (2016). Generalized Statistical Mechanics Description of Fault and Earthquake Populations in Corinth Rift (Greece). Ph.D. Thesis.

[67] Doutsos N., Kontopoulos D., Frydas (1987). Neotectonic evolution of northwestern continental Greece. Geol. Rudsch.

[68] Clews J.E. (1989). Structural controls on basin evolution: Neogene to Quaternary of the Ionian zone, Western Greece. J. Geol. Soc..

[69] Kiratzi A., Sokos E., Ganas A., Tselentis A., Benetatos C., Roumelioti Z., Serpetsidaki A., Andriopoulos G., Galanis O., Petrou P. (2008). The April 2007 earthquake swarm near Lake Trichonis and implications for active tectonics in western Greece. Tectonophysics.

[70] Evangelidis C.P., Konstantinou K.I., Melis N.S., Charalambakis M., Stavrakakis G.N. (2008). Waveform Relocation and Focal Mechanism Analysis of an Earthquake Swarm in Trichonis Lake, Western Greece. Bull. Seism. Soc. Am..

[71] Sokos E., Pikoulis V., Psarakis E., Lois A. (2017). The april 2007 swarm in trichonis lake using data from a microseismic network. Bull. Geol. Soc. Greece.

[72] Kassaras I., Kapetanidis V., Karakonstantis A., Kaviris G., Papadimitriou P., Voulgaris N., Makropoulos K., Popandopoulos G., Moshou A. (2014). The April–June 2007 Trichonis Lake earthquake swarm (W. Greece): New implications toward the causative fault zone. J. Geodyn..

[73] Peterek A., Schwarze J. (2004). Architecture and Late Pliocene to recent evolution of outer-arc basins of the Hellenic subduction zone (south-central Crete, Greece). J. Geodyn..

[74] Gatsios T., Cigna F., Tapete D., Sakkas V., Pavlou K., Parcharidis I. (2020). Copernicus Sentinel-1 MT-InSAR, GNSS and Seismic Monitoring of Deformation Patterns and Trends at the Methana Volcano, Greece. Appl. Sci..

[75] Tibaldi A., Pasquarè F., Papanikolaou D., Nomikou P. (2008). Tectonics of Nisyros Island, Greece, by field and offshore data, and analogue modelling. J. Struct. Geol..

[76] Kaviris G., Kapetanidis V., Spingos I., Sakellariou N., Karakonstantis A., Kouskouna V., Elias P., Karavias A., Sakkas V., Gatsios T. (2022). Investigation of the Thiva 2020–2021 Earthquake Sequence Using Seismological Data and Space Techniques. Appl. Sci..

[77] Ambraseys N.N., Jackson J.A. (1997). Seismicity and strain in the gulf of corinth (greece) since 1694. J. Earthq. Eng..

[78] Ambraseys N.N., Jackson J.A. (1990). Seismicity and associated strain of central Greece between 1890 and 1988. Geophys. J. Int..

[79] Ganas A., Oikonomou I.A., Tsimi C. (2017). NOAfaults: A digital database for active faults in Greece. Bull. Geol. Soc. Greece.

[80] Wyss M., Wiemer S., Zuniga R. (2001). Zmap: A Tool for Analyses of Seismicity Patterns, Typical Applications and Uses: A Cookbook. https://www.researchgate.net/publication/261170802.

[81] Wiemer S. (2000). Minimum Magnitude of Completeness in Earthquake Catalogs: Examples from Alaska, the Western United States, and Japan. Bull. Seism. Soc. Am..

[82] Papadakis G., Vallianatos F., Sammonds P. (2016). Non-extensive statistical physics applied to heat flow and the earthquake frequency–magnitude distribution in Greece. Phys. A Stat. Mech. Appl..

[83] Vallianatos F., Michas G., Papadakis G., Sammonds P. (2012). A non-extensive statistical physics view to the spatiotemporal properties of the June 1995, Aigion earthquake (M6.2) aftershock sequence (West Corinth rift, Greece). Acta Geophys..

[84] Chochlaki K., Vallianatos F., Michas G. (2018). Global regionalized seismicity in view of Non-Extensive Statistical Physics. Phys. A Stat. Mech. Appl..

[85] Telesca L. (2010). Non-extensivity in seismicity. The case of L’Aquila area (central Italy), struck by the April 6th 2009 earthquake (ML5.8). EGU Gen. Assem. Conf. Abstr..

[86] Chelidze T., Matcharashvili T. (2007). Complexity of seismic process; measuring and applications—A review. Tectonophysics.

[87] Matcharashvili T., Chelidze T., Javakhishvili Z., Jorjiashvili N., Paleo U.F. (2011). Non-extensive statistical analysis of seismicity in the area of Javakheti, Georgia. Comput. Geosci..

[88] Darooneh A.H., Dadashinia C. (2008). Analysis of the spatial and temporal distributions between successive earthquakes: Nonextensive statistical mechanics viewpoint. Phys. A Stat. Mech. Appl..

[89] Hasumi T. (2007). Interoccurrence time statistics in the two-dimensional Burridge-Knopoff earthquake model. Phys. Rev. E.

[90] Hasumi T. (2009). Hypocenter interval statistics between successive earthquakes in the two-dimensional Burridge–Knopoff model. Phys. A Stat. Mech. Appl..

[91] Papadakis G., Vallianatos F., Sammonds P. (2013). Evidence of Nonextensive Statistical Physics behavior of the Hellenic Subduction Zone seismicity. Tectonophysics.

[92] Ogata Y. (1998). Space-Time Point-Process Models for Earthquake Occurrences. Ann. Inst. Stat. Math..

[93] Anyfadi E.-A., Avgerinou S.-E., Michas G., Vallianatos F. (2022). Universal Non-Extensive Statistical Physics Temporal Pattern of Major Subduction Zone Aftershock Sequences. Entropy.

[94] Avgerinou S.-E., Anyfadi E.-A., Michas G., Vallianatos F. (2023). A Non-Extensive Statistical Physics View of the Temporal Properties of the Recent Aftershock Sequences of Strong Earthquakes in Greece. Appl. Sci..

[95] Vallianatos F., Michas G. (2020). Complexity of Fracturing in Terms of Non-Extensive Statistical Physics: From Earthquake Faults to Arctic Sea Ice Fracturing. Entropy.

[96] Vallianatos F. (2011). A non-extensive statistical physics approach to the polarity reversals of the geomagnetic field. Phys. A Stat. Mech. Appl..

[97] Vallianatos F., Michas G., Papadakis G. (2018). Nonextensive Statistical Seismology. Complexity of Seismic Time Series.

[98] Chatzopoulos G. (2019). Geodynamic and Seismological Investigation of the South Hellenic Arc Structure.

